# Aspirin-Induced Massive Spontaneous Sublingual Hematoma

**DOI:** 10.7759/cureus.60684

**Published:** 2024-05-20

**Authors:** Utkal P Mishra, Ganakalyan Behera, Prince Handa, Rohit Singh, Fasin Nawas

**Affiliations:** 1 Otolaryngology - Head and Neck Surgery, All India Institute of Medical Sciences, Bhopal, IND

**Keywords:** anticoagulant therapy, antiplatelet therapy, conservative management, airway obstruction, spontaneous, aspirin, sublingual hematoma

## Abstract

Sublingual hematoma, a rare but potentially life-threatening condition, can arise spontaneously or secondary to various triggers, including trauma, dental procedures, or anticoagulant therapy. We present a case of massive spontaneous sublingual hematoma in a 45-year-old woman receiving aspirin therapy for rheumatic heart disease. Despite the absence of trauma or procedural triggers, the patient presented with bleeding from the floor of the mouth and significant submental swelling, prompting urgent intervention to secure the airway and manage coagulopathy. Conservative measures, including discontinuation of aspirin and intravenous vitamin K administration, led to gradual hematoma resolution and favorable patient outcomes. This case highlights the importance of prompt recognition and early management of sublingual hematoma, particularly in the context of aspirin therapy-induced coagulopathy.

## Introduction

Sublingual hematoma is a rare condition characterized by the accumulation of blood beneath the mucous membrane of the floor of the mouth. It typically arises from various factors such as trauma, dental procedures, intraoral suturing, biopsies from the floor of the mouth, tongue bites, bleeding disorders, and as a complication of anticoagulant therapy [1]. In rare instances, sublingual hematoma can happen spontaneously without any triggering factors that may lead to significant swelling in the mouth, potentially causing airway obstruction and other associated complications.

Aspirin, also known as acetylsalicylic acid (ASA), is a widely used medication with antiplatelet and anti-inflammatory properties. It is commonly prescribed for the secondary prevention of cardiovascular events such as stroke and myocardial infarction [2]. The efficacy of aspirin in reducing the risk of vascular events in patients with atherothrombotic disease is well-established, making it a key component in the management of cardiovascular conditions. Despite its benefits, the common adverse events related to aspirin include gastrointestinal ulcers and bleeding [3]. In this report, we describe the presentation and management of a rare case of massive spontaneous sublingual hematoma secondary to aspirin therapy.

## Case presentation

A 45-year-old lady presented to the emergency department with a one-day history of spontaneous bleeding from the floor of the mouth, accompanied by rapid swelling in the submental region. She also reported symptoms of dysphagia and slurred speech. She had a medical history significant for rheumatic heart disease with moderate mitral stenosis, mild tricuspid regurgitation, and hypothyroidism. Three months prior, she underwent mitral valve repair involving mitral commissurotomy and left atrium clot removal. At the time of presentation, she was receiving aspirin 75 mg, metoprolol 25 mg, and thyroxine 12.5 mcg. There was no history of trauma, dental procedures, or violent coughing, and no associated difficulty in breathing, epistaxis, melena, or hematuria.

**Figure 1:**
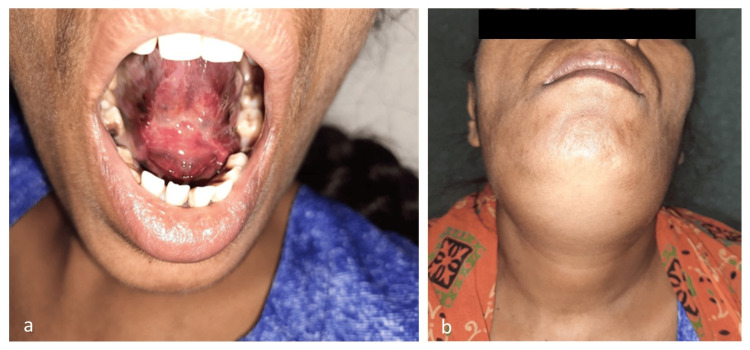
Clinical images on the day of presentation: (a) hematoma of the floor of the mouth, (b) swelling of the submental region

Clinical examination revealed a 4x4 cm firm, non-tender swelling in the submental region with normal overlying skin. On intraoral examination, a large purple-red, non-tender, and soft swelling was noted on the floor of the mouth (Figure 1). The swelling was found to be displacing the tongue superiorly with restriction of tongue movement. Fiber optic laryngoscopy revealed diffuse ecchymosis and edema of the vallecula, the lingual surface of the epiglottis, and aryepiglottic folds. Both the vocal cords and glottic inlet were within normal limits. There was no evident respiratory distress or stridor. Ultrasonographic evaluation of the submental region revealed a 4.5x4 cm hypoechoic collection in the submental region. Her blood parameters revealed severe anemia (hemoglobin level: 6.6 g/dL), leukocytosis (total leukocyte count: 11670/mm³), mildly decreased platelet count (3.82 lacs/l), and significantly prolonged prothrombin time (16.10 seconds) with a markedly elevated international normalized ratio (INR) of 4.17, indicative of a critical bleeding state and potential coagulopathy.

**Figure 2:**
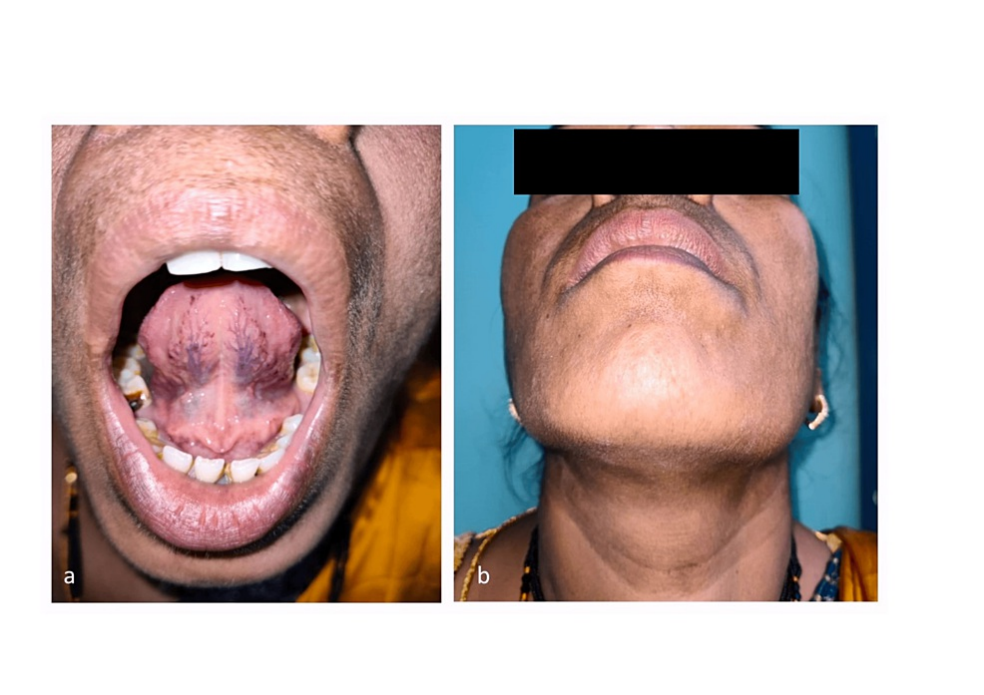
Clinical image after seven days showing (a) resolution of hematoma from the floor of the mouth and (b) resolution of submental swelling

She was transferred to a high-dependency unit for vigilant airway monitoring, with a tracheostomy tray prepared as a precautionary measure. We stopped aspirin and administered 10mg of intravenous vitamin K once daily for seven days to reverse the coagulopathy alongside a regimen of trypsin (96 mg), bromelain (180 mg), and rutoside (200 mg) thrice daily. She underwent regular flexible fiber optic laryngoscopic evaluations to monitor the progression of the hematoma. Both intraoral and submental swelling subsided gradually, and the patient was discharged in stable condition on the seventh day (Figure 2). A follow-up visit after three months revealed her to be in good health, with no signs of recurrence of sublingual swelling or hematoma.

## Discussion

Bleeding due to coagulopathy is a major complication associated with anticoagulation therapy. Aspirin (acetylsalicylic acid) is one of the most commonly used antiplatelet drugs in the world. In low doses (75 to 81 mg/day), it irreversibly inhibits cyclooxygenase -1(COX-1) enzyme by acetylation of serine residue of COX-1 [2]. This mechanism confers an antithrombotic effect, yet paradoxically, it precipitates in a fivefold increase in the incidence of major bleeding events necessitating hospitalization [2,3]. Aspirin-induced coagulopathy usually manifests as gastrointestinal bleeding or epistaxis.

The sublingual space is situated beneath the tongue and superior to the mylohyoid muscle; the sublingual space harbors vital neurovascular structures, including the lingual artery and vein, sublingual gland, and submandibular duct. Hemorrhage within this confined space can precipitate rapid hematoma expansion, leading to elevation of the tongue and floor of the mouth, thereby encroaching upon the upper airway and posing a risk of airway compromise similar to Ludwig's angina.

Most of the previously reported cases of sublingual hematoma occurred as a result of submucosal bleeding triggered by maxillofacial trauma, tongue trauma, tongue bite following a seizure episode, bleeding disorders, and warfarin-induced coagulopathy [4,5]. Coagulopathy-induced sublingual hematoma occurs due to the rupture of atherosclerotic lingual vessels supplying floor-of-mouth musculatures [6].

Common signs and symptoms of sublingual hematoma encompass a spectrum of clinical presentations indicative of upper airway compromise and hemorrhagic involvement. Patients typically present with noticeable drooling of saliva, dysphagia, throat discomfort, dyspnea, and alterations in voice quality. On clinical examination, findings such as a raised floor of the mouth and tongue, ecchymosis, sublingual swelling, cervical mass, retropharyngeal bulge, tachypnea, and stridor are frequently encountered. In more severe cases, sublingual hematomas exhibit rapid progression and can extend into the larynx, causing airway compromise that is often manifested as inspiratory stridor.

In the management of sublingual hematoma, the primary aim is to secure the airway, as airway compromise can be a life-threatening complication. Studies have suggested that procedures such as tracheostomy, cricothyroidectomy, and fiber optic intubation should be preferred over endotracheal intubation in cases of airway obstruction [4]. In our case, as there were no signs of airway compromise, we chose a conservative approach of management with serial fiber optic laryngoscopic evaluation with tracheostomy on standby. Previous cases of warfarin-induced coagulopathy resulting in sublingual hematoma have been managed conservatively using interventions like vitamin K, fresh frozen plasma, and prothrombin complex concentrates [5]. Some cases even utilized leeches to prevent clot formation in the hematoma [7]. In the presented case, only vitamin K and dexamethasone were used to prevent the progression of tongue edema and further airway compromise. The debate over surgical drainage versus conservative management continues, with many cases resolving spontaneously once the underlying cause is addressed [8]. Surgical drainage carries risks such as excessive bleeding, and its efficacy remains uncertain. Individualized decision-making is crucial, as larger progressive hematomas may necessitate drainage to alleviate airway compromise.

## Conclusions

In conclusion, the management of sublingual hematoma induced by aspirin therapy requires a multidisciplinary approach focusing on securing the airway and addressing the underlying coagulopathy. Conservative measures such as discontinuation of aspirin, reversal of coagulopathy with vitamin K, and close monitoring of the airway can be effective in cases without immediate airway compromise. The decision between surgical drainage and conservative management should be individualized based on the size and progression of the hematoma to prevent potential airway compromise.
